# The Association Between Patients' eHealth Literacy and Satisfaction With Shared Decision-making and Well-being: Multicenter Cross-sectional Study

**DOI:** 10.2196/26721

**Published:** 2021-09-24

**Authors:** Richard Huan Xu, Ling-Ming Zhou, Eliza Lai-Yi Wong, Dong Wang

**Affiliations:** 1 Department of Rehabilitation Sciences The Hong Kong Polytechnic University Hong Kong Hong Kong; 2 JC School of Public Health and Primary Care The Chinese University of Hong Kong Hong Kong Hong Kong; 3 School of Health Management Southern Medical University Guangzhou China

**Keywords:** eHealth literacy, shared decision-making, well-being, eHEALS, ICECAP-A

## Abstract

**Background:**

Although previous studies have shown that a high level of health literacy can improve patients’ ability to engage in health-related shared decision-making (SDM) and improve their quality of life, few studies have investigated the role of eHealth literacy in improving patient satisfaction with SDM (SSDM) and well-being.

**Objective:**

This study aims to assess the relationship between patients’ eHealth literacy and their socioeconomic determinants and to investigate the association between patients’ eHealth literacy and their SSDM and well-being.

**Methods:**

The data used in this study were obtained from a multicenter cross-sectional survey in China. The eHealth Literacy Scale (eHEALS) and Investigating Choice Experiments Capability Measure for Adults were used to measure patients’ eHealth literacy and capability well-being, respectively. The SSDM was assessed by using a self-administered questionnaire. The Kruskal-Wallis one-way analysis of variance and Wilcoxon signed-rank test were used to compare the differences in the eHEALS, SSDM, and Investigating Choice Experiments Capability Measure for Adults scores of patients with varying background characteristics. Ordinary least square regression models were used to assess the relationship among eHealth literacy, SSDM, and well-being adjusted by patients’ background characteristics.

**Results:**

A total of 569 patients completed the questionnaire. Patients who were male, were highly educated, were childless, were fully employed, were without chronic conditions, and indicated no depressive disorder reported a higher mean score on the eHEALS. Younger patients (SSDM_≥61 years_=88.6 vs SSDM_16-30 years_=84.2) tended to show higher SSDM. Patients who were rural residents and were well paid were more likely to report good capability well-being. Patients who had a higher SSDM and better capability well-being reported a significantly higher level of eHealth literacy than those who had lower SSDM and poorer capability well-being. The regression models showed a positive relationship between eHealth literacy and both SSDM (*β*=.22; *P*<.001) and well-being (*β*=.26; *P*<.001) after adjusting for patients’ demographic, socioeconomic status, lifestyle, and health status variables.

**Conclusions:**

This study showed that patients with a high level of eHealth literacy are more likely to experience optimal SDM and improved capability well-being. However, patients’ depressive status may alter the relationship between eHealth literacy and SSDM.

## Introduction

### Background

eHealth literacy refers to the acquisition and use of web-based information and communication technology to make appropriate health decisions [1]. Unlike collecting health information through traditional methods (eg, hospital pamphlets and medical magazines), acquiring information from the internet requires extended skills [2-4]. For this task, people need to have professional knowledge about specific health issues, computer and mobile phone literacy, knowledge and skills to navigate the internet, and the ability to analyze and digest web-based information [5,6]. In recent decades, the rapid proliferation of web-based information about health and health care has significantly changed individuals’ health-seeking behaviors, such as participating in web-based communities or purchasing products on the internet and services to improve health and well-being. The internet increasingly serves as a major source of health information for individuals to understand their health concerns, instead of seeking professional advice [2,7].

The internet provides a convenient way to approach health-related information to the public; however, a low level of eHealth literacy may lead, in contrast, to serious harm [8] and health-related social inequality [9,10]. Previous studies have shown that individuals with a low level of eHealth literacy are more likely to report insufficient use of preventive health services [11], negative health-related attitudes [12], unhealthy behaviors and lifestyles [13], and poor medical adherence [14]. However, globally, the relationship between patients’ eHealth literacy and well-being remains insufficiently explored. Nabi et al [15] indicated that seeking information from social networks (eg, Facebook) impacts people’s stress levels and, in turn, influences their physical and psychological well-being. Another systematic review found that providing breast cancer patients with access to digital systems or technological devices could improve their health and well-being [16]. Given the complex and fragmented nature of the current health care systems and the high prevalence of chronic conditions, the internet has been increasingly identified as an essential and valuable information source to support patient-centered care and help patients and their families seek cost-effective health care services [17].

Recently, shared decision-making (SDM) has been reported to be an effective way to improve trust in patient–doctor relationships, reduce negative emotions, and promote patients’ well-being [18,19]. eHealth literacy, as an important concept rooted in the practice of patient-centered care, is increasingly suggested to be used to improve SDM in clinical practice. For example, Nejati et al [20] found that low levels of eHealth literacy can limit patients’ trust in the health care system and their communication patterns and are a barrier to patient participation in the decision-making process. As the global population becomes increasingly reliant on the internet to locate and obtain health information and services [21,22], patients and their caregivers struggle to possess adequate eHealth literacy to engage in the decision-making process. The limited availability of web-based health information restricts patients from participating in their health care decision-making process. For example, Car et al [23] pointed out that poor eHealth literacy limited patients’ ability to make decisions in medicine management. Netjati et al [20] also showed that lower levels of eHealth literacy are associated with poorer SDM among patients with multiple myeloma. However, in China, there is no evidence regarding the relationship between eHealth literacy and medical decision-making.

As reported, there are more than 980 million internet users in China, accounting for more than 20% of the users worldwide [24]. In 2018, the State Council of China, jointly with the National Health Commission, released a series of decrees to encourage integration of traditional industries with internet technologies to improve the quality and efficiency of health care services [25]. A new nationwide web-based service system will be developed to provide patients with a novel way to approach quality health care information and facilitate their active engagement in clinical decision-making. This could help health care providers not only in understanding patients’ preferences, needs, and satisfaction but also in clarifying their health care situations, treatment options, and likely outcomes [26]. Thus, an individual’s level of eHealth literacy is the key to searching and using internet-based health care services to improve their health outcomes and well-being [27]. In 2020, the COVID-19 pandemic further proved that innovative eHealth approaches are vital for delivering health services and supporting patients to prevent contracting COVID-19 and increase their willingness to get vaccinated [28,29]. However, in China, there is a dearth of information regarding patients’ level of eHealth literacy and whether eHealth literacy could improve their satisfaction with SDM (SSDM) and well-being in clinical practice. Without adequate information about these associations, there is a risk that internet-based interventions may lead to some negative outcomes, such as producing a digital divide, solidifying current health disparities, and perpetuating inequities, all of which could result in poor health outcomes and well-being [21].

### Objectives

This study aims to (1) assess the relationship between patients’ eHealth literacy and their socioeconomic status (SES) and (2) investigate the association between patients’ eHealth literacy and their SSDM and well-being.

## Methods

### Study Design and Data Collection

The data used in this study were obtained from a multicenter cross-sectional survey that investigated patients’ attitudes toward patient-centered care (PCC) in Guangdong province, China, from November 2019 to January 2020. Patients were recruited from the inpatient departments of 8 hospitals from 5 cities (Guangzhou, Shenzhen, Zhanjiang, Meizhou, and Shaoguan). All patients from the target hospitals were invited to participate in the survey during the survey period. The inclusion criteria were as follows: (1) being aged ≥18 years, (2) being able to read and speak Chinese, (3) having no cognitive impairment, and (4) being able to provide informed consent. With the assistance of ward nurses, all eligible patients were invited to participate in the survey. The patients who agreed to participate in the survey and provided written informed consent were asked to complete a structured questionnaire that included questions about their demographic characteristics, SES, health conditions, well-being, use of health services, lifestyle, and attitudes toward PCC. A convenience sample of 569 patients (569/800, 71.1% response rate) successfully completed the questionnaire and provided valid responses. The study protocol and informed consent were approved by the institutional review board of the Second Affiliated Hospital of Guangzhou Medical University (reference ID: 2019-ks-28).

### Measures

#### eHealth Literacy

The eHealth Literacy Scale (eHEALS) was used to measure consumers’ combined knowledge, comfort, and perceived skills at finding, evaluating, and applying eHealth information to manage health problems [30]. It was developed based on a framework that comprised six dimensions to understand and use eHealth information [31]. The eHEALS has eight items that are rated on a 5-point Likert scale (including “strongly disagree,” “disagree,” “neutral,” “agree,” and “strongly agree”). The sum score ranges from 8 to 40, where a higher score indicates greater perceived eHealth literacy. To compare it with the results of other measures, in this study, the eHEALS sum score was converted to an overall score between 0 and 100 based on minimum-maximum normalization. A simplified Chinese eHEALS was used in this study [32].

#### Well-being

The Investigating Choice Experiments Capability Measure for Adults (ICECAP-A) is a generic and preference-based instrument that evaluates an individual’s capability well-being [33]. Each dimension of the ICECAP-A comprises 1 item with 4 response options that range from “not capable” to “fully capable,” to measure the different aspects related to capability well-being. The results of the ICECAP-A can convert to a summarized utility score that ranges from 0 to 1 to support the economic evaluation of social care interventions [34]. In this study, the ICECAP-A sum score was calculated using the scoring formula provided by the University of Birmingham. To facilitate comparability with the other measures, in this study, we converted the original ICECAP-A utility score to a range between 0 and 100. The Chinese version of the ICECAP-A was used [35].

#### Patient SSDM

Patient SSDM was assessed using a self-administered questionnaire. It was developed based on our previous patient engagement framework [36] and index [37], findings from literature review, focus group interviews (including patients, doctors, nurses, and policy makers), and expert discussion. It assessed patient satisfaction with decision-making, along with doctors in clinical practice. The SSDM comprises 5 items to measure different dimensions of satisfaction with the SDM. They are (1) “Did doctors provide several selections for you when making decisions (selection),” (2) “Did doctors carefully listen to your health problems when making decisions (Listen),” (3) “Did doctors respect your willingness when making choices (Respect),” (4) “Did doctors fully discuss your concerns with you when making decisions (Discussion),” and (5) “Did doctors fully understand your preferences and needs when making decisions (preference)?” Each item was rated on a 5-point scale ranging from “strongly disagree” to “strongly agree.” The instrument showed good content, construct (Multimedia Appendix 1), convergent validity (Multimedia Appendix 2), and high internal consistency reliability (Cronbach α=.93). The overall score of the SSDM was calculated by adding up the scores of each item, which were then converted to a range of 0 to 100 based on minimum-maximum normalization.

#### Depressive Disorder

The Patient Health Questionnaire-2 was used to assess whether patients experienced depressed mood over the past 2 weeks. An individual with a score of 3 or above (range: 0-6) was recognized as someone with a depressive disorder [38].

### Statistical Analysis

Patients’ background characteristics (section 1: demographics; section 2: SES; section 3: lifestyle; and section 4: health status) were presented with the mean and SD of the eHEALS, SSDM, and ICECAP-A sum scores. The Kruskal-Wallis one-way analysis of variance (multiple groups) and Wilcoxon signed-rank test (2 groups) were used to compare the differences in the eHEALS, SSDM, and ICECAP-A sum scores of patients with different background characteristics. The Wilcoxon signed-rank test was also used to assess the relationship between level of eHealth literacy and SSDM and well-being. Patients’ level of eHealth literacy was recategorized into high (≥30) and low (<30) on the basis of the median of the original eHEALS sum score. In addition, patients’ depressive status was considered in the analysis of the relationship between 3 measures. Three ordinary least square multivariate regression models were developed to assess the relationships between measures adjusted by patients’ background characteristics. In the first model, the dependent variable was eHealth literacy, and the independent variables were SSDM, well-being, and patients’ background characteristics. In the second model, SSDM was the dependent variable, and the independent variables were eHealth literacy and patients’ background characteristics. In the third model, the dependent variable was capability well-being, and the independent variables were eHealth literacy and patients’ background characteristics. The objective of the first model was to assess the relationship between patients’ eHealth literacy and their socioeconomic determinants, whereas the other two models assessed how patients’ eHealth literacy can predict the changes in their SSDM and well-being, after adjusting for background characteristics. The Bland-Altman (B-A) plot was used to assess the agreement between three measures. The mean scores of the eHEALS, SSDM, and ICECAP-A were plotted on the x-axis, and the differences between them was plotted on the y-axis. The observations clustered evenly around a horizontal line representing y=0, reflecting good agreement between the measures [39]. R software (R Foundation for Statistical Computing) was used to perform all statistical analyses. The level of statistical significance was set at *P*≤.05.

## Results

### Participants’ Characteristics and the Results of Measures

Table 1 shows that more than half of the patients were male (288/569, 50.6%), and approximately 18.6% (106/569) and 19.2% (109/569) were aged <30 years and >60 years, respectively. Nearly 51.1% (291/569) of the patients indicated living with at least one kind of chronic condition, and 27.9% (159/569) reported having a depressive disorder. Patients who were male (eHEALS_male_=68.5 vs eHEALS_female_=64.3), were highly educated (eHEALS_tertiary_=69.6 vs eHEALS_no or primary_=62.9), were childless (eHEALS_no child_=72.7 vs eHEALS_with child_=59.4), fully employed (eHEALS_fully employed_=68.5 vs eHEALS_nonemployed_=61.7), were without chronic conditions (eHEALS_no chronic conditions_=68.5 vs eHEALS_with chronic conditions_=64.4), and indicated no depressive disorder (eHEALS_no depression_=67.6 vs eHEALS_with depression_=63.3) reported a higher level of eHealth literacy. Younger patients (SSDM_≥61 years_=88.6 vs SSDM_16-30 years_=84.2) tended to show higher SSDM. Patients, who resided in rural areas (ICECAP-A_rural_=79.3 vs ICECAP-A_urban_=75.7) and were well paid (ICECAP-A_≥Chinese ¥6401 (US $960.15)_=81.9 vs ICECAP-A_≤Chinese ¥1800 (US $270)_=73.5) were highly likely to report better capability well-being.

**Table 1:** Patients’ characteristics and scores of the eHEALS^a^, SSDM^b^, and ICECAP-A^c^.

			Patients, n (%)		eHEALS					SSDM							ICECAP-A			
					Value, mean (SD)		*P* value^d^			Value, mean (SD)			*P* value			Value, mean (SD)			*P* value	
Overall			569 (100)		66.4 (21.2)		—^e^			85.7 (17.0)			—			77.5 (15.8)			—	
**Sex**								.02						.55						.14
	Female	281 (49.4)		64.3 (21.4)					85.3 (17.5)						79.8 (16.2)					
	Male	288 (50.6)		68.5 (21)					86.1 (16.6)						83.6 (15.3)					
**Age (years)**								.003						.03						.85
	16-30	106 (18.6)		71.7 (19.9)					84.2 (18.8)						77.9 (15)					
	31-40	132 (23.2)		69.6 (18.2)					84.1 (16.3)						78.1 (15.7)					
	41-50	116 (20.4)		66.0 (19.4)					84.4 (17.5)						78.4 (15.3)					
	51-60	106 (18.6)		63.7 (22.4)					87.5 (18.3)						76.8 (15.4)					
	≥61	109 (19.2)		60.5 (24.7)					88.6 (13.9)						76.3 (17.6)					
**Education**								.01						.40						<.001
	No or primary	90 (15.8)		62.9 (25.2)					87.8 (15)						70.9 (19.7)					
	Secondary	215 (37.8)		64.0 (22.1)					84.8 (18.4)						75.6 (16.1)					
	Tertiary or above	264 (46.4)		69.6 (18.5)					85.7 (16.5)						81.3 (12.9)					
**Marital status**								.03						.70						.92
	Single	95 (16.7)		72.5 (17.9)					84.5 (17.5)						78.7 (13.6)					
	Married	446 (78.4)		65.1 (21.8)					85.8 (17)						77.3 (15.9)					
	Divorced, widow, or widower	28 (4.9)		67.1 (19.9)					87.0 (16.3)						75.8 (20.3)					
**Family registry**								.86						.35						.03
	Rural	279 (49.1)		66.5 (20.3)					86.5 (16.3)						79.3 (14.1)					
	urban	290 (50.9)		66.3 (22.2)					84.8 (17.7)						75.7 (17.2)					
**Number of children**								.02						.52						.05
	0	104 (18.3)		72.7 (17.8)					84 (18.5)						78.9 (14.3)					
	1	170 (29.9)		66.4 (21.3)					86.7 (15)						79.5 (15.3)					
	2	202 (35.5)		66.5 (20.4)					85.4 (16.4)						75.7 (16.3)					
	≥3	93 (16.3)		59.4 (24.3)					86.4 (20.0)						76.1 (16.7)					
**Caregiver**								.42						.31						.48
	No	414 (72.8)		65.8 (21.7)					85.4 (17.1)						77.1 (16.2)					
	Yes	155 (27.2)		68 (20.0)					86.4 (16.9)						78.5 (14.7)					
**Living status**								.86						.41						.98
	Live with family or others	512 (89.9)		66.4 (21.4)					85.6 (16.9)						77.4 (16)					
	Live alone	57 (10.1)		66.6 (20.4)					86.2 (18.6)						78.3 (13.8)					
**Employment status**								.005						.11						.29
	Employed	394 (69.2)		68.5 (20.2)					85.1 (17.1)						78.1 (15.1)					
	Nonemployed	175 (30.8)		61.7 (22.8)					87.1 (16.9)						76.1 (17.3)					
**Disposable income per month (Chinese ¥ [US $])**								.55						.62						<.001
	≤1800 (270)	155 (27.2)		64.7 (22.6)					84.9 (18.4)						73.5 (18.1)					
	1801-3800 (270.15-570)	146 (25.7)		65.5 (22.9)					87.4 (15.4)						76.6 (15.8)					
	3801-6400 (570.15-960)	127 (22.3)		68.7 (18.2)					85.5 (16.3)						78.6 (14.0)					
	≥6401 (960.15)	141 (24.8)		67.3 (20.3)					84.9 (17.7)						81.9 (13.4)					
**Health insurance**								.97						.98						.93
	FHS^f^	30 (5.3)		68.3 (18.9)					87.3 (14.9)						81.1 (11.7)					
	UEBMI^g^	258 (45.3)		67 (20.3)					85.3 (17.2)						78.3 (14.3)					
	URBMI^h^	132 (23.2)		65.6 (21.8)					85.8 (18.4)						75.8 (18.7)					
	NRCMS^i^	131 (23)		65.7 (22.9)					85.7 (16.4)						76.9 (16.0)					
	No	18 (3.2)		67.2 (23.4)					87.7 (12.5)						77.2 (16.6)					
**BMI^j^**								.79						.03						.03
	Normal	242 (42.5)		66.5 (21.9)					83.7 (18.4)						75.7 (17.1)					
	Abnormal	327 (57.5)		66.3 (20.8)					87.1 (15.8)						78.9 (14.6)					
**Smoking**								.04						.17						.71
	No	408 (71.7)		70.2 (21.0)					84.9 (17.3)						77.7 (15.5)					
	Sometimes	83 (14.5)		69 (21.2)					85.6 (12.0)						76.9 (18.3)					
	Everyday	78 (13.8)		65.2 (21.9)					89.7 (19.4)						76.9 (14.3)					
**Healthy diet per week**								.20						.49						.23
	Few	87 (15.3)		65.4 (21.1)					84.8 (20.2)						74.4 (18.0)					
	Sometimes	310 (54.5)		66.4 (20.8)					85.1 (16.9)						77.8 (15.0)					
	Everyday	172 (30.2)		70.2 (21.9)					87.2 (15.5)						78.6 (15.1)					
**Exercise per week**								.009						.28						.004
	Never	151 (26.5)		63.2 (23.7)					84.3 (17.8)						73.2 (19.0)					
	Sometimes	321 (56.4)		68.8 (20.2)					86.1 (16.3)						78.6 (14.0)					
	Always	97 (17.1)		63.5 (19.6)					86.5 (18.2)						80.7 (14.6)					
**Chronic condition**								.008						.56						.003
	No	278 (48.9)		68.5 (20.9)					85 (17.6)						79.3 (15.5)					
	Yes	291 (51.1)		64.4 (21.4)					86.3 (16.4)						75.7 (15.9)					
**Depressive disorder**								.04						.07						<.001
	No	410 (72.1)		67.6 (20.9)					86.3 (16.8)						80.6 (13.8)					
	Yes	159 (27.9)		63.3 (21.9)					84 (17.5)						69.4 (17.7)					
**Self-reported health condition**								.50						.11						.08
	Severe threat to life	113 (19.9)		66.6 (22.1)					88 (16.3)						73.9 (17.3)					
	Moderate threat to life	113 (19.9)		64 (22.1)					83.6 (18.4)						77.7 (15.4)					
	Mild threat to life	136 (23.9)		65.6 (19.4)					85.6 (15.5)						78.1 (14.2)					
	No threat to life	207 (36.3)		68.2 (21.4)					85.6 (17.5)						78.9 (16.0)					

^a^eHEALS: eHealth Literacy Scale.

^b^SSDM: satisfaction with shared decision-making.

^c^ICECAP-A: Investigating Choice Experiments Capability Measure for Adults.

^d^*P* value was calculated based on a Kruskal-Wallis one-way analysis of variance (multiple groups) and Wilcoxon signed-rank test (2 groups).

^e^Not available.

^f^FHS: free health care scheme.

^g^UEBMI: urban employee basic medical insurance.

^h^URBMI: urban resident basic medical insurance.

^i^NRCMS: new rural cooperative medical care system.

^j^BMI: normal: 18.5≤BMI<23; abnormal: BMI<18.5 or BMI≥23.

### Relationship Among eHealth Literacy, SSDM, and Well-being

Table 2 presents the outcomes of SSDM and well-being in patients with different levels of eHealth literacy. Patients with a higher level of eHealth literacy reported higher SSDM and better well-being than those with a lower level of eHealth literacy. For patients with depressive disorder, the difference in SSDM in patients with different levels of eHealth literacy was statistically nonsignificant.

**Table 2:** Satisfaction with SDM^a^ and well-being in different groups of eHealth literacy and stratified by patients’ depressive disorder and chronic condition status.

		Satisfaction with SDM	Well-being
**Overall**			
	High eHealth literacy, mean (SD)	88.7 (14.7)	81.1 (14.7)
	Low eHealth literacy, mean (SD)	82.4 (18.7)	73.7 (16.1)
	*P* value^b^	<.001	<.001
**With depressive disorder**			
	High eHealth literacy, mean (SD)	86.2(16.2)	73.3(18)
	Low eHealth literacy, mean (SD)	82.2 (18.4)	66.1 (16.8)
	*P* value	.10	.004
**Without depressive disorder**			
	High eHealth literacy, mean (SD)	89.5 (14.2)	83.6 (12.4)
	Low eHealth literacy, mean (SD)	82.5 (18.9)	77.1 (14.6)
	*P* value	<.001	<.001
**With chronic conditions**			
	High eHealth literacy, mean (SD)	88.4 (14.7)	82.6 (14.4)
	Low eHealth literacy, mean (SD)	80.5 (20.1)	75 (15.8)
	*P* value	<.001	<.001
**Without chronic conditions**			
	High eHealth literacy, mean (SD)	89 (14.8)	79.1 (14.8)
	Low eHealth literacy, mean (SD)	83.9 (17.5)	72.7 (16.4)
	*P* value	<.001	<.001

^a^SDM: shared decision-making.

^b^*P* value was calculated based on Wilcoxon signed-rank test.

### Results of the Regression Analysis

The results of multivariate regression models showed that there was a significant and positive relationship between eHealth literacy and SSDM and well-being after adjusting for patients’ background characteristics (Table 3). Model 1 demonstrated that patients who were living alone (*β*=−6.82; *P*=.03) and nonemployed (*β*=−4.55; *P*=.02) showed a lower level of eHealth literacy. Models 2 and 3 showed that, after adjustment, eHealth literacy was a statistically significant factor predicting the change in SSDM (*β*=.17; *P*<.001) and well-being (*β*=.15; *P*<.001), respectively. There was a positive relationship between patients’ well-being and their educational level, income, and depressive status.

**Table 3:** Regression analysis of eHealth literacy and satisfaction with shared decision-making (SSDM) and well-being^a^.

Variables		*β* (95% CI)					
		Model 1 (DV^b^=eHEALS^c^)	*P* value	Model 2 (DV=SSDM)	*P* value	Model 3 (DV=ICECAP-A^d^)	*P* value
eHealth literacy		—^e^	—	.17 (0.11 to 0.24)	<.001	.15 (0.09 to 0.21)	<.001
Satisfaction in SDM		.22 (0.12 to 0.32)	<.001	—	—	—	—
Well-being		.26 (0.14 to 0.38)	<.001	—	—	—	—
Sex (male)		2.99 (−1.11 to 7.1)	.15	−1.58 (−5.03 to 1.87)	.37	.29 (−2.67 to 3.25)	.85
**Age (years)**							
	31-40	−2.05 (−8.87 to 4.78)	.56	.68 (−5.03 to 6.4)	.81	2.58 (−2.32 to 7.49)	.30
	41-50	−4.96 (−12.24 to 2.33)	.18	.58 (−5.52 to 6.67)	.85	4.98 (−0.25 to 10.22)	.06
	51-60	−6.37 (−14.15 to 1.41)	.11	4.46 (−2.05 to 10.97)	.18	5.2 (−0.39 to 10.79)	.07
	≥61	−7.91 (−16.35 to 0.53)	.07	5.97 (−1.09 to 13.03)	.1	5.88 (−0.18 to 11.94)	.06
**Education**							
	Secondary	−2.78 (−8.35 to 2.78)	.33	−2.67 (−7.31 to 1.97)	.26	4.51 (0.53 to 8.49)	.03
	Tertiary or above	−.7 (−7.23 to 5.83)	.83	−.91 (−6.33 to 4.5)	.74	8.34 (3.7 to 12.99)	.004
**Marital status**							
	Married	−2.63 (−12.44 to 7.17)	.60	−2.26 (−10.48 to 5.97)	.59	−.35 (−7.41 to 6.71)	.92
	Divorced, widow, or widower	3.57 (−8.77 to 15.91)	.57	−4.92 (−15.26 to 5.42)	.35	.94 (−7.94 to 9.81)	.84
Urban resident		−1.8 (−6.18 to 2.59)	.42	−.3 (−3.98 to 3.38)	.87	−.07 (−3.23 to 3.09)	.97
**Children**							
	1	−2.84 (−13.15 to 7.47)	.59	4.02 (−4.62 to 12.66)	.36	.01 (−7.41 to 7.43)	.99
	2	−1.06 (−11.56 to 9.43)	.84	2.77 (−6.03 to 11.56)	.54	−2.25 (−9.79 to 5.3)	.56
	≥3	−6.89 (−18.22 to 4.43)	.23	2.87 (−6.63 to 12.38)	.55	1.29 (−6.87 to 9.45)	.76
Caregiver (yes)		.1 (−3.8 to 4.16)	.93	1.33 (−2 to 4.67)	.43	−.04 (−2.9 to 2.82)	.98
Live alone		−6.82 (−13.28 to −0.36)	.04	2.91 (−2.52 to 8.34)	.29	1.35 (−3.31 to 6.02)	.57
Nonemployed		−4.55 (−9.02 to −0.08)	.04	1.43 (−2.33 to 5.19)	.45	.35 (−2.88 to 3.57)	.83
**Income (Chinese ¥ [US $])**							
	1801-3800 (270.15-570)	−1.34 (−6.23 to 3.54)	.59	2.05 (−2.04 to 6.15)	.32	1.94 (−1.58 to 5.45)	.28
	3801-6400 (570.15-960)	−1.32 (−6.9 to 4.25)	.64	1.13 (−3.54 to 5.8)	.63	2.43 (−1.58 to 6.44)	.23
	≥6401 (960.15)	−4.74 (−10.69 to 1.21)	.12	.47 (−4.51 to 5.45)	.85	4.84 (0.56 to 9.11)	.03
**Insurance**							
	Urban employee basic medical insurance	−3.03 (−10.82 to 4.76)	.45	−2.17 (−8.7 to 4.35)	.51	−2.48 (−8.08 to 3.13)	.39
	Urban resident basic medical insurance	−2.21 (−10.47 to 6.06)	.60	−1.53 (−8.47 to 5.4)	.66	−2.07 (−8.02 to 3.88)	.50
	New rural cooperative medical care system	−2.12 (−10.91 to 6.67)	.64	−1.91 (−9.28 to 5.46)	.61	−.02 (−6.35 to 6.31)	.99
	No	−4.95 (−17.27 to 7.38)	.43	.66 (−9.68 to 11)	.90	2.05 (−6.82 to 10.93)	.65
BMI (abnormal)		−2.29 (−5.78 to 1.19)	.20	3.17 (0.26 to 6.08)	.03	2.38 (−0.12 to 4.87)	.06
**Smoking status**							
	Sometimes	2.56 (−2.83 to 7.96)	.35	6.04 (1.54 to 10.54)	.01	.01 (−3.85 to 3.87)	.99
	Everyday	2.84 (−2.8 to 8.48)	.32	2.13 (−2.6 to 6.86)	.38	−1.49 (−5.55 to 2.57)	.47
**Healthy diet**							
	Sometimes	−6.28 (−11.19 to −1.38)	.01	.79 (−3.35 to 4.92)	.71	2.37 (−1.18 to 5.91)	.19
	Everyday	−2.45 (−7.92 to 3.01)	.38	2.15 (−2.42 to 6.73)	.36	2.61 (−1.31 to 6.54)	.19
**Exercise**							
	Sometimes	4.13 (0.02 to 8.24)	.04	1.43 (−2.01 to 4.87)	.41	3.7 (0.74 to 6.65)	.01
	Always	−1.94 (−7.36 to 3.47)	.48	1.89 (−2.63 to 6.4)	.41	4.9 (1.03 to 8.77)	.01
Chronic condition (yes)		.07 (−3.6 to 3.75)	.97	1.12 (−1.95 to 4.19)	.47	−2.5 (−5.14 to 0.13)	.06
Depressive disorder (yes)		−1.6 (−5.62 to 2.42)	.43	−1.94 (−5.2 to 1.32)	.24	−8.55 (−11.35 to −5.75)	<.001
Moderate threat to life		−2.84 (−8.24 to 2.55)	.30	−3.41 (−7.92 to 1.1)	.14	2.71 (−1.16 to 6.58)	.17
Mild threat to life		−2.62 (−7.93 to 2.69)	.33	−1.76 (−6.21 to 2.69)	.44	.64 (−3.18 to 4.47)	.74
No threat to life		−.62 (−5.66 to 4.42)	.81	−1.34 (−5.57 to 2.89)	.53	.1 (−3.53 to 3.73)	.96

^a^Reference: female, 16-30 years, no or primary education, single, rural resident, no child, no caregiver, live with family or others, income ≤ Chinese ¥1800 (US $270), free health care scheme insurance, normal BMI, no smoking, few healthy diets, no exercise, no chronic conditions, no depressive disorder, and severe threat to life.

^b^DV: dependent variable.

^c^eHEALS: eHealth Literacy Scale.

^d^ICECAP-A: Investigating Choice Experiments Capability Measure for Adults.

^e^Not available.

### Agreement Between Measures

**Figure 1:**
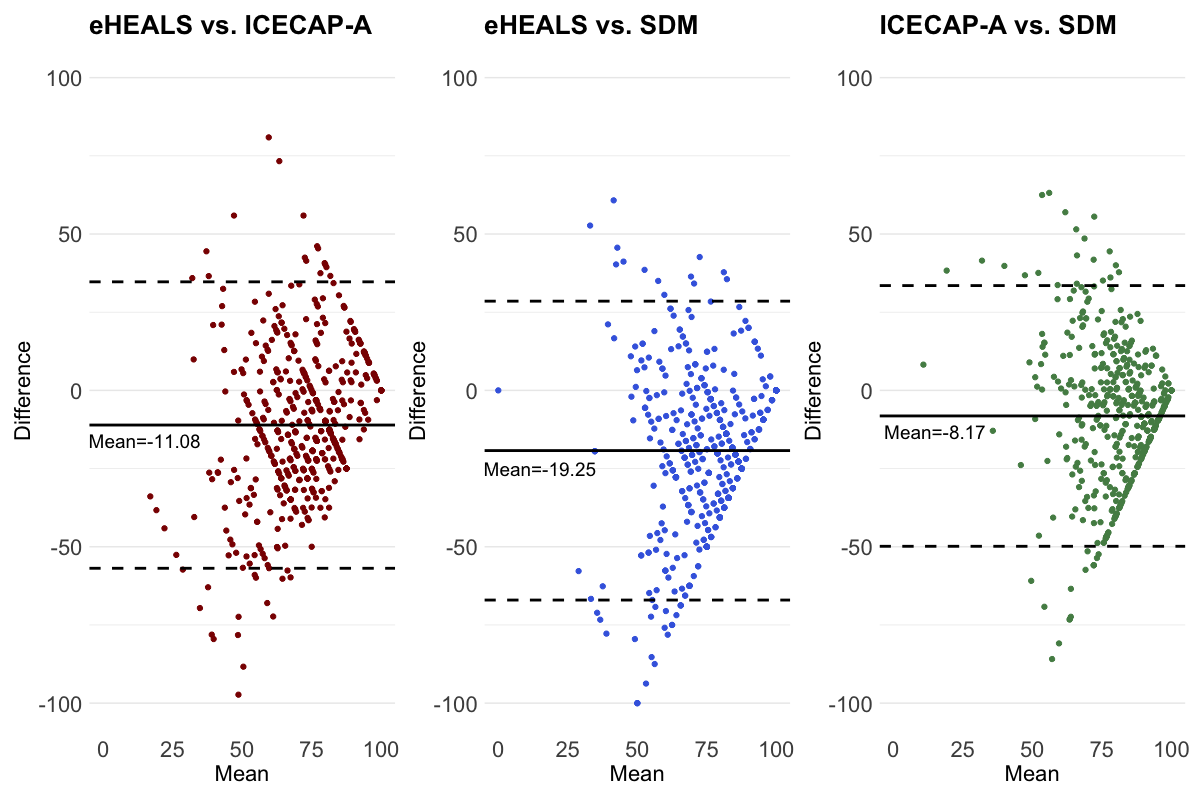
Agreement between scores of the eHEALS, satisfaction with SDM, and ICECAP-A. eHEALS: eHealth Literacy Scale; ICECAP-A: Investigating Choice Experiments Capability Measure for Adults; SDM: shared decision-making.

Although the B-A plot shows a wide limit of agreement interval between the three measures, systematic differences were detected. A good agreement was observed in patients who reported a high level of eHealth literacy, SSDM, and well-being; however, patients who reported a low level of eHealth literacy, SSDM, and well-being were more likely to show less consistent results across the measures, indicating low agreement (Figure 1).

## Discussion

### Principal Findings

This study extended the findings of previous studies by demonstrating a statistically significant association between eHealth literacy and SSDM and capability well-being in a sample of Chinese patients. However, when patients reported a low level of eHealth literacy, its association with SSDM and well-being turned to weak and inconsistent. Our findings suggested that providing training to improve patients’ eHealth literacy may be a useful way to strengthen their ability to search and use web-based health and health care information to improve their activity in clinical decision-making and well-being. However, although the internet carries a vast range of information resources and services to help people manage their health, we noticed that disparities in using the internet are persistent in people with low SES (unemployed status and unhealthy lifestyle) and, therefore, affect their potential to maintain and improve eHealth literacy and limit their ability to navigate the health care system. In addition, there seemed to be a negative relationship between patients’ mental health status and their use of internet-based knowledge and skills to improve SSDM. However, further research is needed to support this finding, as it has not been studied extensively.

### Comparisons With Previous Studies

Our results firstly exhibited that there is a positive relationship between eHealth literacy and Chinese patients’ SSDM, which is in line with the findings of previous studies. For example, an Iranian study indicated that eHealth literacy is positively associated with SDM and patient communication patterns in patients with multiple myeloma [20]. Another German study noted that the regular use of eHealth services facilitated the decision-making process for patients with cancer and their families [40]. Other studies have reported that the internet and web-based courses are fundamental in improving patients’ communication skills with medical personnel [3,41], reducing their overall medical expenses [42], and increasing their confidence and knowledge to be involved in decision-making [43]. In addition, we found that patients who reported being very satisfied with SDM (satisfied with all five dimensions of the SSDM) obtained a similar mean score on the eHEALS (Multimedia Appendix 3). This indicated that improving eHealth literacy might be a multifaceted strategy to promote all the existential dimensions of the SDM [44] and, in turn, positively associated with patients’ physical, psychological, social, and spiritual well-being [45]. Considering that the internet is increasingly serving as a major source of health information for both medical professionals and patients, improving eHealth literacy could be a cost-effective way to transfer the current paternalistic pattern of medical services to a more patient-centric model [2]. Moreover, doctors’ attitude toward SDM was identified as another important factor that supported SDM in improving patients’ well-being in clinical practice [46], which should be considered for future studies.

Studies examining the relationship between patients’ well-being and eHealth-related interventions have recently been explored. For example, Villani et al [47] indicated that eHealth interventions can significantly reduce emotional suppression of patients with cancer and increase their cancer-related emotional well-being. Pagliari [48] and Patel et al [49] found that the proliferation of personal health information technology has enhanced people’s ability to manage their own health care, communicate with providers through social networks, meet their informational needs, search patient educational resources, and make preferred health decisions. However, research examining the role of eHealth literacy in using these techniques to improve patients’ well-being is in its infancy. This study found that patients who reported a high level of eHealth literacy were more likely to show full capability well-being than those who reported a low level of eHealth literacy. This supports the notion that patients with high levels of eHealth literacy are confident and capable of handling internet-based tools to improve their health and well-being [21,22,50]. Further, unlike studies that used nonpreference-based instruments to measure well-being, in this study, the ICECAP-A, a preference-based measure, generated outcomes that not only reflect a patient’s current well-being status but also provide information to support the estimation of social care–related quality of life and facilitate a cost-effectiveness analysis of eHealth-related interventions and policies [33]. Furthermore, the results of the B-A plot exhibited a poor agreement between eHEALS, SSDM, and ICECAP-A when patients reported a low score on those measures, unlike those who reported a high score. Methodologically, this may be because, in this study, few patients reported having low well-being and unsatisfactory SDM, and less than 19.8% (113/569) of the respondents reported having poor health. Thus, we could not validate our findings in these populations. Due to the cross-sectional design, no causal relationships can be concluded. Therefore, further studies are required.

The results of bivariate analysis indicated that patients with high SES and healthy lifestyle are more likely to indicate a high level of eHealth literacy; however, the multivariable regression analysis showed a different picture. This is consistent with the mixed findings of the relationship between individuals’ socioeconomic determinants and their level of eHealth literacy, as reported in previous studies. For example, Lwin et al [51] found that women and men did not differ in their reported frequencies of evaluating eHealth information; however, no older adult respondents (>55 years) were involved in their study. Conversely, a study in China found that female respondents showed a higher level of eHealth literacy than male respondents; however, they used a revised version of the eHEALS, and all the respondents were older than 45 years [52]. In addition, Stellefson et al [53] indicated that women and older adults living with chronic obstructive pulmonary disease showed a low level of eHealth literacy in the United States. Wong and Cheung [54] also indicated that older primary care service users in Hong Kong are highly likely to report low levels of eHealth literacy. The findings of multivariable regression analysis showed that, when adjusted for patients’ background characteristics, there was no significant relationship between patients’ eHealth literacy and their educational level. This suggests that eHealth literacy does not comprise only basic literacy but also an accumulation of knowledge and skills to navigate the internet to use health care services. Norman [31] noted that eHealth literacy is not just a combination of the capability to use computers and traditional health literacy but is a meta-literacy comprising different facets of literacy. For example, an undereducated patient with chronic conditions may show higher eHealth literacy than highly educated patients who have recently been diagnosed with cancer. Our findings highlight the importance of promoting eHealth literacy.

When patients reported having depressive disorders, the difference in SSDM between those with high and low eHealth literacy was statistically insignificant. This is not inconsistent with previous findings in those patients with good skills in searching, assessing, and correctly using web-based health care information may lead to decreased levels of hospitalization-related mental disorders and improve their long-term quality of life and well-being [55-57]. However, no study has directly investigated the relationship between eHealth literacy and SDM considering the potential effect of patients’ mental health status. Neter and Brainin [11] confirmed that there is insufficient evidence on the association between eHealth literacy and emotional states of anxiety and depression. A US-based study indicated that social media could benefit patients with chronic obstructive pulmonary disease by helping them cope with mental health issues such as anxiety and depression [53]. Another study found that African Americans who researched depression, anxiety, and stress on the internet showed a significantly higher mean score of the eHEALS than those who did not [58]. Interventions focused on internet-related health literacy, such as mental eHealth literacy, require further investigation. Furthermore, in this study, depressive status was assessed using the Patient Health Questionnaire-2, the sensitivity of which has been disputed by some previous studies [59,60]. Other mental disorders, such as anxiety and stress, should be evaluated in future studies.

### Limitations

It is important to address the limitations of this study. First, this was a cross-sectional study; thus, no causal relationships could be concluded. Second, all the respondents were recruited from inpatient departments in hospitals; the issue of a single information source may affect the validity of our findings. In addition, compared with the data from the 2019 Guangdong census, our respondents were slightly older and comprised a higher proportion of rural residents (Multimedia Appendix 4). This implies that there was some degree of selection bias, which may affect the generalizability of our findings. Third, we did not assess the associations between eHealth literacy, well-being, and SDM stratified by patient disease groups, which might also affect the generalizability of our findings. Fourth, the ICECAP-A score was not estimated based on the preference weight of the Chinese population, which is currently unavailable. This may have affected the validity of our findings. Fifth, all the information was self-reported by the patients, which may have generated recall bias. Finally, the information of patients who refused to participate in the survey was not recorded, which might have led to a degree of information bias.

### Conclusions

According to the findings of this study, patients with a high level of eHealth literacy were more likely to experience an optimal SDM and improved capability well-being. This suggests that the implementation of interventions to strengthen patients’ eHealth literacy could improve their optimal use of health care services and the efficiency of the health and social care system. In addition, univariable analysis demonstrated that patients with low SES showed insufficient eHealth literacy, which may affect their ability to buffer against the negative impacts of an adverse event on their health. It is important for policy makers to understand the facilitators and barriers to improve patients’ eHealth literacy and to develop strategies to enhance their health behaviors and health outcomes. Moreover, the effects of patients’ mental health status on the relationship between eHealth literacy and SSDM require further investigation.

## Appendix

Multimedia Appendix 1 Results of the confirmatory factor analysis of the satisfaction with shared decision-making.

Multimedia Appendix 2 Correlation between the satisfaction with shared decision-making and 9-item Shared Decision-Making Questionnaire.

Multimedia Appendix 3 eHEALS scores stratified by items of the ICECAP-A and satisfaction with SDM. eHEALS: eHealth Literacy Scale; ICECAP-A: Investigating Choice Experiments Capability Measure for Adults; SDM: shared decision-making. *<italic>P</italic><.05; **<italic>P</italic><.01; ***<italic>P</italic><.001

Multimedia Appendix 4 Comparisons between the sample and Guangdong general population.

## Abbreviations

B-A: Bland-Altman
eHEALS: eHealth Literacy Scale
ICECAP-A: Investigating Choice Experiments Capability Measure for Adults
SDM: shared decision-making
SES: socioeconomic status
SSDM: satisfaction with shared decision-making

## References

[1] Neter E, Brainin E (2012). eHealth literacy: extending the digital divide to the realm of health information. J Med Internet Res.

[2] Paige SR, Krieger JL, Stellefson M, Alber JM (2017). eHealth literacy in chronic disease patients: an item response theory analysis of the eHealth literacy scale (eHEALS). Patient Educ Couns.

[3] Silver MP (2015). Patient perspectives on online health information and communication with doctors: a qualitative study of patients 50 years old and over. J Med Internet Res.

[4] Karnoe A, Furstrand D, Christensen KB, Norgaard O, Kayser L (2018). Assessing competencies needed to engage with digital health services: development of the eHealth literacy assessment toolkit. J Med Internet Res.

[5] van der Vaart R, van Deursen AJ, Drossaert CH, Taal E, van Dijk JA, van de Laar MA (2011). Does the eHealth Literacy Scale (eHEALS) measure what it intends to measure? Validation of a Dutch version of the eHEALS in two adult populations. J Med Internet Res.

[6] Parker S, Prince A, Thomas L, Song H, Milosevic D, Harris MF, IMPACT Study Group (2018). Electronic, mobile and telehealth tools for vulnerable patients with chronic disease: a systematic review and realist synthesis. BMJ Open.

[7] Quinn S, Bond R, Nugent C (2017). Quantifying health literacy and eHealth literacy using existing instruments and browser-based software for tracking online health information seeking behavior. Comput Hum Behav.

[8] George C, Whitehouse D, Duquenoy P (2012). eHealth: Legal, Ethical and Governance Challenges.

[9] Hansen AH, Bradway M, Broz J, Claudi T, Henriksen Ø, Wangberg SC, Årsand E (2019). Inequalities in the use of eHealth between socioeconomic groups among patients with type 1 and type 2 diabetes: cross-sectional study. J Med Internet Res.

[10] Latulippe K, Hamel C, Giroux D (2017). Social health inequalities and eHealth: a literature review with qualitative synthesis of theoretical and empirical studies. J Med Internet Res.

[11] Neter E, Brainin E (2019). Association between health literacy, eHealth literacy, and health outcomes among patients with long-term conditions: a systematic review. Eur Psychol.

[12] Xie B (2011). Effects of an eHealth literacy intervention for older adults. J Med Internet Res.

[13] Hsu W, Chiang C, Yang S (2014). The effect of individual factors on health behaviors among college students: the mediating effects of eHealth literacy. J Med Internet Res.

[14] Lin C, Ganji M, Griffiths MD, Bravell ME, Broström A, Pakpour AH (2020). Mediated effects of insomnia, psychological distress and medication adherence in the association of eHealth literacy and cardiac events among Iranian older patients with heart failure: a longitudinal study. Eur J Cardiovasc Nurs.

[15] Nabi RL, Prestin A, So J (2013). Facebook friends with (health) benefits? Exploring social network site use and perceptions of social support, stress, and well-being. Cyberpsychol Behav Soc Netw.

[16] Triberti S, Savioni L, Sebri V, Pravettoni G (2019). eHealth for improving quality of life in breast cancer patients: A systematic review. Cancer Treat Rev.

[17] Koay K, Schofield P, Jefford M (2012). Importance of health literacy in oncology. Asia Pac J Clin Oncol.

[18] Brehaut JC, O'Connor AM, Wood TJ, Hack TF, Siminoff L, Gordon E, Feldman-Stewart D (2003). Validation of a decision regret scale. Med Decis Making.

[19] Vahdat S, Hamzehgardeshi L, Hessam S, Hamzehgardeshi Z (2014). Patient involvement in health care decision making: a review. Iran Red Crescent Med J.

[20] Nejati B, Lin C, Aaronson NK, Cheng AS, Browall M, Lin C, Broström A, Pakpour AH (2019). Determinants of satisfactory patient communication and shared decision making in patients with multiple myeloma. Psychooncology.

[21] Tennant B, Stellefson M, Dodd V, Chaney B, Chaney D, Paige S, Alber J (2015). eHealth literacy and Web 2.0 health information seeking behaviors among baby boomers and older adults. J Med Internet Res.

[22] Bodie GD, Dutta MJ (2008). Understanding health literacy for strategic health marketing: eHealth literacy, health disparities, and the digital divide. Health Mark Q.

[23] Car J, Tan WS, Huang Z, Sloot P, Franklin BD (2017). eHealth in the future of medications management: personalisation, monitoring and adherence. BMC Med.

[24] (2021). Number of internet users in China from 2008 to 2020. Statista.

[25] Guideline on the development and promotion of providing internet-based healthcare services in China. http://www.gov.cn/zhengce/content/2018-04/28/content_5286645.htm.

[26] Brennan PF, Strombom I (1998). Improving health care by understanding patient preferences: the role of computer technology. J Am Med Inform Assoc.

[27] (2018). Guideline on the development and promotion of providing internet-based healthcare services in China. http://www.gov.cn/zhengce/content/2018-04/28/content_5286645.htm.

[28] Brørs G, Norman CD, Norekvål TM (2020). Accelerated importance of eHealth literacy in the COVID-19 outbreak and beyond. Eur J Cardiovasc Nurs.

[29] Dong D, Xu RH, Wong EL, Hung C, Feng D, Feng Z, Yeoh E, Wong SY (2020). Public preference for COVID-19 vaccines in China: a discrete choice experiment. Health Expect.

[30] Norman CD, Skinner HA (2006). eHEALS: the eHealth literacy scale. J Med Internet Res.

[31] Norman CD, Skinner HA (2006). eHealth literacy: essential skills for consumer health in a networked world. J Med Internet Res.

[32] Xu RH, Zhou L, Lu SY, Wong EL, Chang J, Wang D (2020). Psychometric validation and cultural adaptation of the simplified Chinese eHealth literacy scale: cross-sectional study. J Med Internet Res.

[33] Al-Janabi H, Flynn TN, Coast J (2012). Development of a self-report measure of capability wellbeing for adults: the ICECAP-A. Qual Life Res.

[34] Flynn TN, Huynh E, Peters TJ, Al-Janabi H, Clemens S, Moody A, Coast J (2015). Scoring the Icecap-a capability instrument. Estimation of a UK general population tariff. Health Econ.

[35] Tang C, Xiong Y, Wu H, Xu J (2018). Adaptation and assessments of the Chinese version of the ICECAP-A measurement. Health Qual Life Outcomes.

[36] Wong EL, Xu RH, Lui S, Cheung AW, Yeoh E (2018). Development of conceptual framework from the view of patients and professionals on patient engagement: a qualitative study in Hong Kong SAR, China. Open J Nurs.

[37] Xu RH, Cheung AW, Wong EL (2018). Development and validation of an instrument to measure patient engagement in Hong Kong Special Administrative Region, China. Patient Prefer Adherence.

[38] Gilbody S, Richards D, Brealey S, Hewitt C (2007). Screening for depression in medical settings with the Patient Health Questionnaire (PHQ): a diagnostic meta-analysis. J Gen Intern Med.

[39] Giavarina D (2015). Understanding Bland Altman analysis. Biochem Med (Zagreb).

[40] Halwas N, Griebel L, Huebner J (2017). eHealth literacy, Internet and eHealth service usage: a survey among cancer patients and their relatives. J Cancer Res Clin Oncol.

[41] Lu X, Zhang R (2019). Impact of physician-patient communication in online health communities on patient compliance: cross-sectional questionnaire study. J Med Internet Res.

[42] Veroff D, Marr A, Wennberg DE (2013). Enhanced support for shared decision making reduced costs of care for patients with preference-sensitive conditions. Health Aff (Millwood).

[43] Hoffmann TC, Del Mar C, Santhirapala R, Freeman A (2020). Teaching clinicians shared decision making and risk communication online: an evaluation study. BMJ Evid Based Med.

[44] Gulbrandsen P, Clayman ML, Beach MC, Han PK, Boss EF, Ofstad EH, Elwyn G (2016). Shared decision-making as an existential journey: aiming for restored autonomous capacity. Patient Educ Couns.

[45] Netter E, Brainin E, Baron-Epel O (2018). The third digital divide in the health domain: is internet use for health purposes associated with health benefits?. eHealth: Current Evidence, Promises, Perils and Future Directions.

[46] Stevenson FA, Barry CA, Britten N, Barber N, Bradley CP (2000). Doctor-patient communication about drugs: the evidence for shared decision making. Soc Sci Med.

[47] Villani D, Cognetta C, Repetto C, Serino S, Toniolo D, Scanzi F, Riva G (2018). Promoting emotional well-being in older breast cancer patients: results from an eHealth intervention. Front Psychol.

[48] Pagliari C (2007). Design and evaluation in eHealth: challenges and implications for an interdisciplinary field. J Med Internet Res.

[49] Patel V, Arocha J, Ancker J (2017). Cognitive Informatics in Health and Biomedicine: Understanding and Modeling Health Behaviors.

[50] Stellefson M, Paige SR, Alber JM, Chaney BH, Chaney D, Apperson A, Mohan A (2019). Association between health literacy, electronic health literacy, disease-specific knowledge, and health-related quality of life among adults with chronic obstructive pulmonary disease: cross-sectional study. J Med Internet Res.

[51] Lwin MO, Panchapakesan C, Sheldenkar A, Calvert GA, Lim LK, Lu J (2020). Determinants of eHealth literacy among adults in China. J Health Commun.

[52] Lin Z, Zhang Y, Matteson M, Li X, Tu X, Zhou Y, Wang J (2020). Older adults’ eHealth literacy and the role libraries can play. J Libr Inf Sci.

[53] Stellefson ML, Shuster JJ, Chaney BH, Paige SR, Alber JM, Chaney JD, Sriram PS (2018). Web-based health information seeking and eHealth literacy among patients living with Chronic Obstructive Pulmonary Disease (COPD). Health Commun.

[54] Wong DK, Cheung M (2019). Online health information seeking and eHealth literacy among patients attending a primary care clinic in Hong Kong: a cross-sectional survey. J Med Internet Res.

[55] Willis E, Royne MB (2017). Online health communities and chronic disease self-management. Health Commun.

[56] Duff OM, Walsh DM, Furlong BA, O'Connor NE, Moran KA, Woods CB (2017). Behavior change techniques in physical activity eHealth interventions for people with cardiovascular disease: systematic review. J Med Internet Res.

[57] Wildevuur SE, Simonse LW (2015). Information and communication technology-enabled person-centered care for the "big five" chronic conditions: scoping review. J Med Internet Res.

[58] James DC, Harville C (2016). Ehealth literacy, online help-seeking behavior, and willingness to participate in mHealth chronic disease research among African Americans, Florida, 2014-2015. Prev Chronic Dis.

[59] Bunevicius A, Deltuva V, Tamasauskas S, Tamasauskas A, Bunevicius R (2013). Screening for psychological distress in neurosurgical brain tumor patients using the Patient Health Questionnaire-2. Psychooncology.

[60] Liu Z, Yu Y, Hu M, Liu H, Zhou L, Xiao S (2016). PHQ-9 and PHQ-2 for screening depression in Chinese rural elderly. PLoS One.

