# Supplementary material for: The Association Between Patients' eHealth Literacy and Satisfaction With Shared Decision-making and Well-being: Multicenter Cross-sectional Study
Source: J Med Internet Res. 2021 Sep 24;23(9):e26721. doi: 10.2196/26721 (PMC8501410; doi:10.2196/26721)
Supplement: Multimedia Appendix 1 [file jmir_v23i9e26721_app1.docx]

**Multimedia Appendix 1.** Results of the confirmatory factor analysis of the satisfaction with shared decision-making.

| **Item** | **Factor loading** | **p-value** |
| --- | --- | --- |
| Selection | 0.8 | <.001 |
| Listen | 0.84 | <.001 |
| Respect | 0.86 | <.001 |
| Discussion | 0.89 | <.001 |
| Preference | 0.85 | <.001 |
| Listen ~~ Respect | 0.3 | <.001 |
|  |  |  |
| **Model fit statistics** | **value** |  |
| Chi-square value | 7.79 |  |
| Degrees of freedom | 4 |  |
| p-value | 0.09 |  |
|  |  |  |
| Comparative fit index | 0.998 |  |
| Tucker-Lewis index | 0.995 |  |
| Root mean square error of approximation | 0.045 |  |
| Standardized root mean square residual | 0.008 |  |
