# Supplementary material for: The Association Between Patients' eHealth Literacy and Satisfaction With Shared Decision-making and Well-being: Multicenter Cross-sectional Study
Source: J Med Internet Res. 2021 Sep 24;23(9):e26721. doi: 10.2196/26721 (PMC8501410; doi:10.2196/26721)
Supplement: Multimedia Appendix 2 [file jmir_v23i9e26721_app2.docx]

**Multimedia Appendix 2.** Correlation between the satisfaction with shared decision-making and 9-item Shared Decision-Making Questionnaire.

|  | **Pearson’s** **correlation** | |
| --- | --- | --- |
|  | **SDM-Q-9** | **p-value** |
| **Selection** | 0.37 | <.001 |
| **Listen** | 0.33 | <.001 |
| **Respect** | 0.34 | <.001 |
| **Discussion** | 0.37 | <.001 |
| **Preference** | 0.38 | <.001 |
| **Sum score** | 0.41 | <.001 |

Note: SDM-Q-9: The 9-item Shared Decision Making Questionnaire;

r>0.3 moderate correlation; r>0.5 strong correlation.
