# Supplementary figures and images for: The Association Between Patients' eHealth Literacy and Satisfaction With Shared Decision-making and Well-being: Multicenter Cross-sectional Study
Source: J Med Internet Res. 2021 Sep 24;23(9):e26721. doi: 10.2196/26721 (PMC8501410; doi:10.2196/26721)

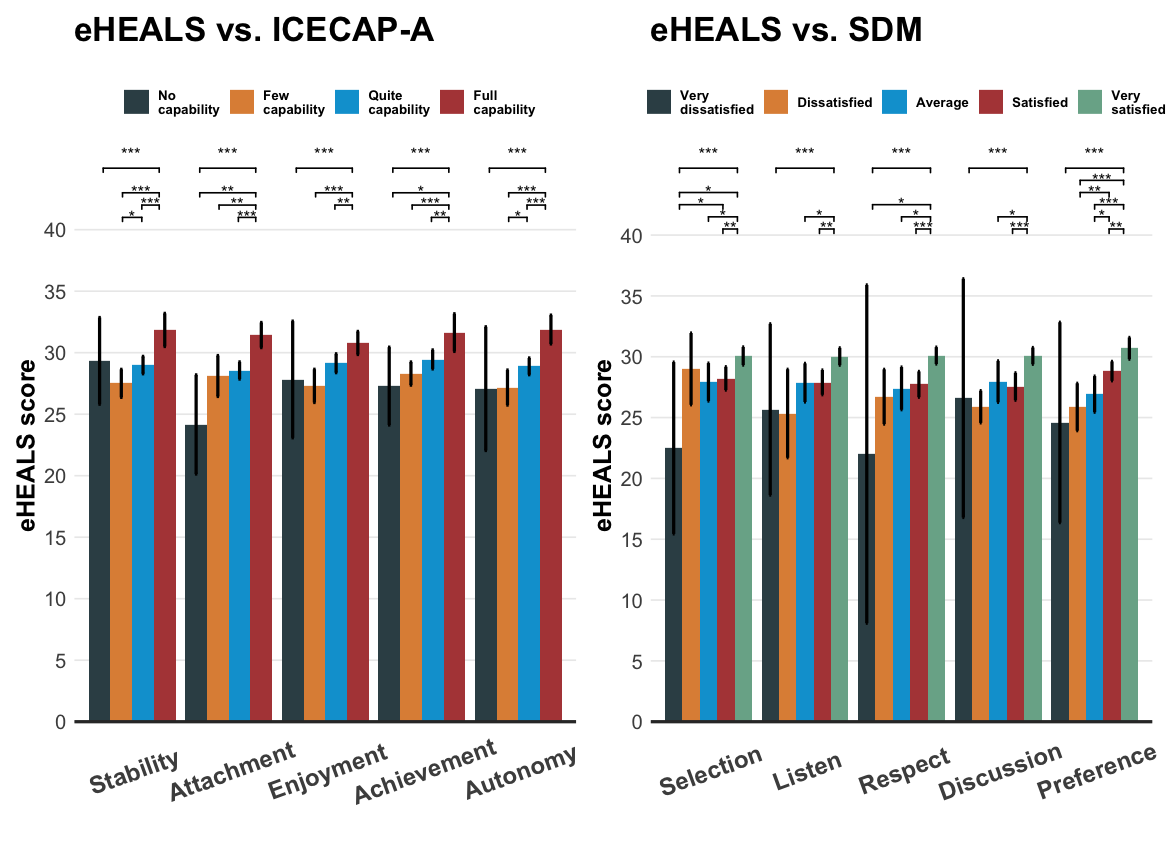

Supplement: Multimedia Appendix 3 [file jmir_v23i9e26721_app3.png]
