# Supplementary material for: The Association Between Patients' eHealth Literacy and Satisfaction With Shared Decision-making and Well-being: Multicenter Cross-sectional Study
Source: J Med Internet Res. 2021 Sep 24;23(9):e26721. doi: 10.2196/26721 (PMC8501410; doi:10.2196/26721)
Supplement: Multimedia Appendix 4 [file jmir_v23i9e26721_app4.docx]

**Multimedia Appendix 4.** Comparisons between the sample and Guangdong general population.

|  | **Sample** | |  | **Guangdong general population** |
| --- | --- | --- | --- | --- |
|  | **n** | **%** |  | **%** |
| **Overall** | 569 | 100 |  |  |
|  |  |  |  |  |
| **Sex** |  |  |  |  |
| **Female** | 281 | 49.3 |  | 47.7 |
| **Male** | 288 | 50.4 |  | 52.3 |
| **Age** |  |  |  |  |
| **0-14** | 0 | 0 |  | 16.3 |
| **15-64** | 488 | 85.7 |  | 74.7 |
| **≥65** | 81 | 14.3 |  | 9 |
| **Family registry** |  |  |  |  |
| **Rural** | 279 | 49.1 |  | 28.6 |
| **urban** | 290 | 50.9 |  | 71.4 |
